# The effects of mechanical force on fibroblast behavior in cutaneous injury

**DOI:** 10.3389/fsurg.2023.1167067

**Published:** 2023-04-18

**Authors:** Charlotte E. Berry, Mauricio Downer, Annah G. Morgan, Michelle Griffin, Norah E. Liang, Lionel Kameni, Jennifer B. Laufey Parker, Jason Guo, Michael T. Longaker, Derrick C. Wan

**Affiliations:** ^1^ Hagey Laboratory for Pediatric Regenerative Medicine, Division of Plastic and Reconstructive Surgery, Department of Surgery, Stanford University School of Medicine, Stanford, CA, United States; ^2^ Institute for Stem Cell Biology and Regenerative Medicine, Stanford University, Stanford, CA, United States

**Keywords:** mechanical force, fibroblast, myofibroblast, scar, review

## Abstract

Wound healing results in the formation of scar tissue which can be associated with functional impairment, psychological stress, and significant socioeconomic cost which exceeds 20 billion dollars annually in the United States alone. Pathologic scarring is often associated with exaggerated action of fibroblasts and subsequent excessive accumulation of extracellular matrix proteins which results in fibrotic thickening of the dermis. In skin wounds, fibroblasts transition to myofibroblasts which contract the wound and contribute to remodeling of the extracellular matrix. Mechanical stress on wounds has long been clinically observed to result in increased pathologic scar formation, and studies over the past decade have begun to uncover the cellular mechanisms that underly this phenomenon. In this article, we will review the investigations which have identified proteins involved in mechano-sensing, such as focal adhesion kinase, as well as other important pathway components that relay the transcriptional effects of mechanical forces, such as RhoA/ROCK, the hippo pathway, YAP/TAZ, and Piezo1. Additionally, we will discuss findings in animal models which show the inhibition of these pathways to promote wound healing, reduce contracture, mitigate scar formation, and restore normal extracellular matrix architecture. Recent advances in single cell RNA sequencing and spatial transcriptomics and the resulting ability to further characterize mechanoresponsive fibroblast subpopulations and the genes that define them will be summarized. Given the importance of mechanical signaling in scar formation, several clinical treatments focused on reducing tension on the wound have been developed and are described here. Finally, we will look toward future research which may reveal novel cellular pathways and deepen our understanding of the pathogenesis of pathologic scarring. The past decade of scientific inquiry has drawn many lines connecting these cellular mechanisms that may lead to a map for the development of transitional treatments for patients on the path to scarless healing.

## Introduction

Pathologic skin scarring is a type of fibroproliferative disorder not dissimilar from the fibrotic response that occurs following injury in many other tissue types. This fibrotic response can be protective for tissues, as a means of re-establishing integrity expeditiously following injury. However, efficiency is frequently traded for full restoration of tissue function in fibrotic healing. Though scaring of the skin is a well-recognized and visible example of this phenomenon, fibrotic response to injury exists throughout the human body (1). Idiopathic pulmonary fibrosis in the lungs, hepatic cirrhosis in the liver, and the response in myocardial tissue to ischemic damage are all examples of this process (1–4). Taken together, fibroproliferative disease accounts for approximately 50% of deaths in United States and the economic cost is estimated to be in the range of tens of billions of dollars (5). Socioeconomically, there is substantial cost of these diseases in terms of disability and impact on quality of life (1).

In the skin, scarring may occur in response to any deep cutaneous injury including trauma, burns, or iatrogenic causes including surgery or radiation therapy. In the developed world, scar-related pathology affects approximately 100 million people (6–8). Scars can result in functional limitations (i.e., contracture), cosmetic concerns, and affect quality of life by causing pain, pruritus, and psychological distress (6, 9).

While superficial wounds may heal without significant scar formation, deep cutaneous injury frequently results in permanent, disfiguring scarring and may cause more significant problems, such as hypertrophic scars or keloids. These two types of pathologic scarring represent examples of abnormal, pathologic wound healing. Pathologic scarring is typically understood as “over-healing,” an overcorrection caused by an exaggerated response of normal wound healing pathways (6, 9, 10). To better define these terms and understand how over-healing occurs, we must first establish the four stages of normal wound healing.

### Normal wound healing

The wound healing process is coordinated by interactions between several cell types, systems, and pathways in the body, all with the goal of re-establishing the skin barrier. Though complex, the process is quite precise and controlled in healthy humans. In the process of normal wound healing, four phases have been identified: hemostasis, inflammation, proliferation, and remodeling. These phases interact and overlap substantially, influencing each other and the entire healing process (11, 12).

Hemostasis, the first phase of the wound healing process, begins immediately after the wound is incurred. The goal of this stage is to attenuate blood loss. Blood flow is slowed through constriction of the blood vessels and, with the activation of both intrinsic and extrinsic coagulation pathways, ultimately stopped by aggregation of platelets and the formation of a fibrin clot (11). The fibrin clot and surrounding wound tissue will secrete proinflammatory cytokines and several growth factors, including transforming growth factor 1, 2, and 3 (TGF-β1, TGF-β2, TGF-β3), platelet-derived growth factor types A, B, C, and D (PDGF-A, PDGF-B, PDGF-C, and PDGF-D), fibroblast growth factor subtypes 2 and 7 (FGF-2, and FGF-7), and epidermal growth factor (EGF), to attract and promote proliferation of immune cells (12, 13).

Inflammation, the second phase, begins once bleeding has ceased. The blood vessels dilate to allow an influx of immune cells, such as M1 macrophages, neutrophils, and lymphocytes into the wound. These cells adhere to the fibrin clot and recruit more inflammatory cells. Neutrophils are responsible for decontaminating the wound by phagocytizing any bacteria and cellular debris found in the wound and releasing reactive oxygen species, producing cytotoxic granules, and placing neutrophil extracellular traps (NETs) (11). Neutrophils additionally amplify inflammation, induce fibroblast proliferation, and direct the adaptive immune response (14). Lymphocytes, like T and B cells, are responsible for fighting off any possible infections and regulating the overall immune response, though the activity of these cells continues into the late proliferation/early remodeling phase (12). Macrophages are involved in many interactions throughout the wound healing process. Initially, pro-inflammatory M1 macrophages are responsible for releasing cytokines that recruit and activate additional leukocytes. Anti-inflammatory M2 macrophages later induce apoptosis in cells that are no longer needed, including other immune cells, allowing for the resolution of the inflammatory phase. As these apoptotic cells are removed, macrophages promote the transition to the proliferation phase by recruiting fibroblasts, keratinocytes, and endothelial cells to begin tissue regeneration (1, 12, 14).

Proliferation, the third phase, is a continuous process that overlaps with the inflammatory phase. The goal of this phase is re-epithelialization of the wounded tissue (11). With respect to the dermis, fibroblasts and endothelial cells are the primary cell types involved in this process. These cells promote formation of collagen, granulation tissue, and angiogenesis (12). Fibroblasts are responsible for collagen synthesis, as well as the production of proteoglycans and glycosaminoglycans, major components of the extracellular matrix that help to stabilize and form a scaffold for the healing tissue (11, 12). Once this foundation has been created, epithelial cells from the wound periphery migrate inwards and layer over the healing wound bed. Concurrently vasculogenesis begins. As the wound begins to mature, collagen fibers are laid down by fibroblasts (11).

Remodeling, the final phase, typically takes months to a year but can continue for years after the initial wounding. The goal of this phase is to return the architecture of the tissue to its unwounded state (12). The new extracellular matrix that was created in the proliferation phase is altered during remodeling. Many of the capillaries formed to deliver blood and cells to newly generated tissue regress and the density of blood vessels decreases to a normal state (11). The collagen that was laid down by fibroblasts is re-organized to better strengthen the healing tissue. Myofibroblasts, or contractile fibroblasts, contract the periphery of the wounded area to reduce the size of the wound (1, 12, 15). Ultimately, the once functional tissue is transformed into a scar composed mainly of fibroblasts and a collagenous extracellular matrix. A fully healed scar will never be as strong as the original tissue, but can recover up to 80% of the tensile strength of the original (11, 15). Remodeling will continue after this scar has formed, organizing and degrading excess collagen in an attempt to return to its original unwounded state (11, 15).

### Excessive wound healing and pathologic scarring

The typical phases of wound healing often result in effective resolution of superficial damage to cutaneous tissue. However, when wounds involve the dermis, scarring may occur. Two primary types of pathologic scarring are recognized: hypertrophic scars and keloids. Though these two processes are differentiated by their pathogenesis, characteristic features, histological morphology, and clinical treatments, differentiating between the two clinically remains challenging.

### Hypertrophic scarring

Hypertrophic scars are clinically defined as erythematous, raised, and often pruritic lesions that are contained within the area of the causative wound. Classically, they appear adjacent to joints in areas of skin which are frequent recipients of tensile force (16, 17). The natural history of a hypertrophic scar is rapid growth for the first 4–12 weeks following injury with flattening and regression during the remodeling phase.

### Keloids

Keloids may result from diverse types of injury to the skin, including but not limited to perforation, laceration, scratches, insect bites, acne, burns, and iatrogenic injuries from surgery. They appear most frequently in areas under tension, such as the neck, chest, shoulders, upper back, and abdomen. Unlike the contained nature of hypertrophic scars, keloids extrude from the site of the initial injury and involve adjacent tissue (18). This fibroproliferative pathology may occur up to years following an initial insult to the tissue and expand at a slower rate than hypertrophic scars. While hypertrophic scars regress, keloids almost always continue to expand without regression and have been likened to benign tumors of the skin (16, 18). Genetic and epigenetic factors appear to play a major role in the pathogenesis of keloids, as they are more common in those with African and Asian ancestry and often run in families (19, 20).

Due to the considerable morbidity, patient well-being, and economic toll incurred by pathologic scarring, significant interest in developing effective clinical therapies exists. While early research has focused on elucidating and targeting the biochemical mechanisms contributing to pathologic scarring, recent interest has centered around the relationship between mechanical forces and scarring (5).

## Mechanical stimulation influences wound healing

**Figure 1:**
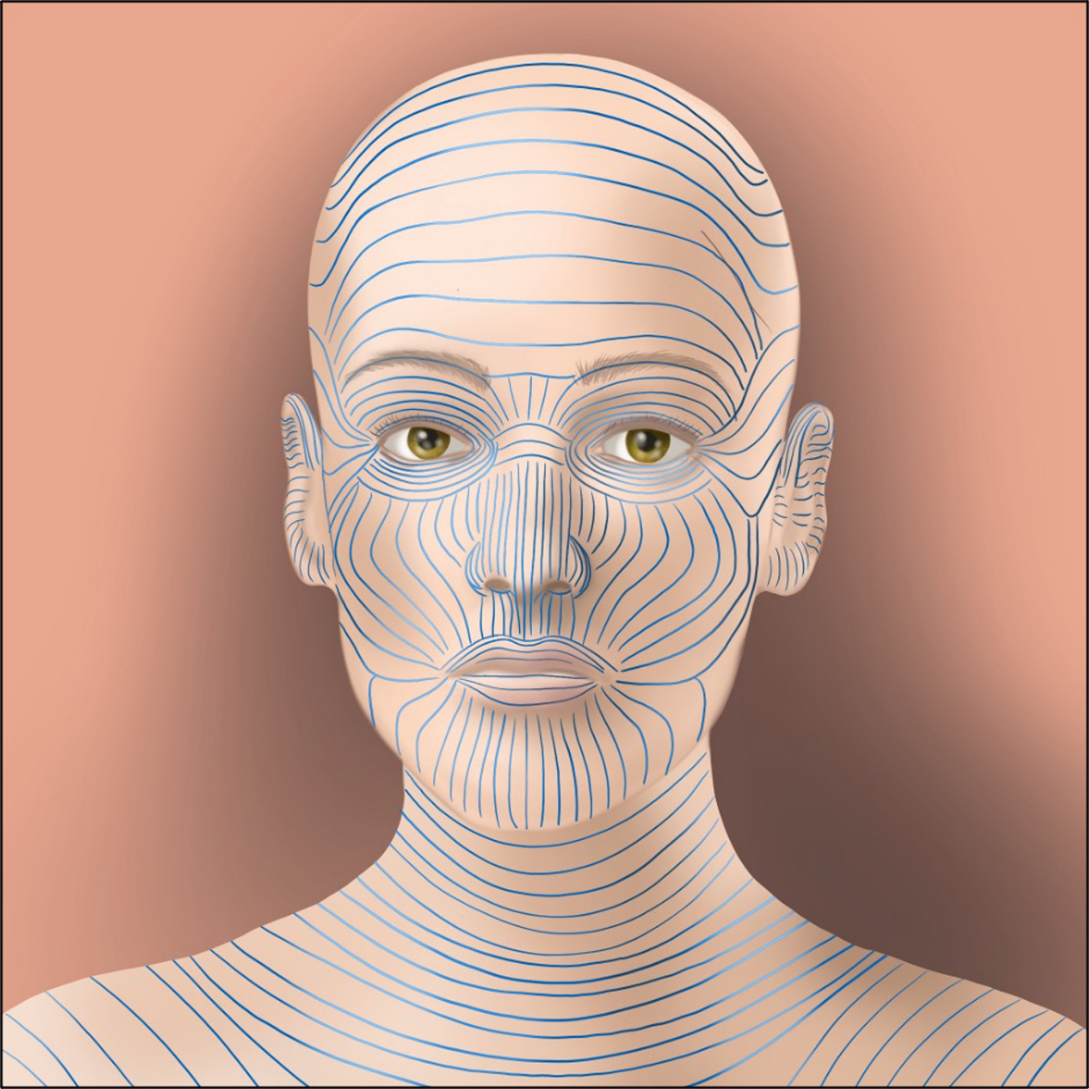
An example of Langer lines, the approximations of tension directionality across the skin surface. These lines have been recapitulated many times and are used to direct the ideal directionality of incisions to minimize tension across a wound, one of the earliest historical responses to the observation that increasing tension aggravates the scarring process.

The association between scar formation and mechanical forces was first observed nearly 200 years ago by Guillaume Dupuytren, the French anatomist and surgeon who observed puncture wounds through the skin produce an elliptical wound in 1834. In 1861, Karl Langer, an Austrian anatomist, utilized this technique in conjunction with topographical skin lines to develop the first set of guidelines to dictate the ideal orientation of surgical incisions (Figure 1) (21). Since that time, over thirty-six guidelines attempting to improve upon Langer's work have been developed, using concepts such as the orientation of underlying muscles and the development of wrinkles in the skin, all with the same goal: to decrease scarring through minimizing anatomical tension across the wound (22).

Recent studies have demonstrated that regional differences in stiffness of the overlying skin may correlate with propensity for scar formation, indicating the existence of anatomically disparate “scar zones” (23). Lack of mechanical load has also been implicated as one contributing factor to the scarless healing phenomenon observed in certain tissue types, such as fetal wounds and oral mucosa (24, 25).

These scar zones may also explain the propensity of keloids to appear at anatomical sites which are subject to increased stiffness and mechanical force, such as the chest and upper back (26, 27). The characteristic shapes of keloids, typically described as a “butterfly,” “crab's claw,” or “dumbbell,” also provide an early suggestion that mechanical force contributes meaningfully to the pathogenesis of this fibroproliferative disorder (28). Computer visual analysis of keloids has since confirmed that the highest tension indeed exists at the hyper-proliferative edges of keloids, rather than the less proliferative center (26).

A 2011 study by Gurtner et al. demonstrated that cutaneous scar formation could be dictated by controlling the mechanical environment of the wound. In a porcine model, wounds were created and allowed to heal under applied tension, normal tension, and tension-offloading. Tension-offloading resulted in a histologic scar area that was reduced by 6-fold compared to the control state, and 9-fold compared to the applied tension state (29). This study, amongst others, has confirmed in a quantitative manner the qualitative observations regarding scar behavior under tension that have long been observed clinically and anecdotally.

## Model systems

As interest has grown in studying the biochemical pathways that underlie transduction of mechanical signals and result in the clinically observed phenomenon of exacerbation of scarring by mechanical force, a number of *in vitro* and *in vivo* model systems have been developed. Both of these model systems provide important, though different, lenses to examine the role of fibroblasts behavior in a mechanically dynamic environment. While *in vitro* systems allow for the examination of single cell types and even single cells at a given time and under highly regulated environments, *in vivo* systems allow for the study of fibroblasts within the complex and interconnected system of a living organism which more closely mirrors a clinical environment.

### In vitro model systems

Fibroblasts, long known to be responsible for fibrotic deposition of excessive extracellular matrix (ECM) in the healing process, were identified early on to adopt a fibroproliferative phenotype following mechanical stimulation. For this reason, most *in vitro* models of mechanical stimulation focus on this cell type.

Just as our understanding of fibroblasts and their behavior in response to mechanical force has expanded significantly in the last century, so too has our methodology in studying it. Early attempts to do so *in vitro* involved primitive “hanging drop” culture methods which created mechanical stimulation by establishing tissue cultures in a droplet and subsequently stretching the culture over a silicone rod during the growth period (30, 31). Since then, iterative attempts to improve upon *in vitro* model systems to be more representative of the biological environment of the fibroblast have been proposed and implemented.

Several *in vitro* models have been produced and studied which include cell types other than fibroblasts, such as Langerhans cells, melanocytes, endothelial cells, and others (32–34). While the goal of these studies has frequently been to create a cellular environment more similar to that of *in vivo* skin, a growing body of literature has come to identify that mechanical force results in morphological and biochemical changes to many non-fibroblast cell types as well (34, 35).

The importance of the ECM and the physical framework in which cells exist were recognized early in the study of mechanical force on fibroblast behavior. Developmental biologist Paul Weiss created experiments utilizing fibroblasts cultured in plasma clot and applied tension to the fibrin to examine resulting morphological changes in the fibroblasts (36). The discovery that fibroblasts could be studied in acid-solubilized collagen gave rise to the movement from two-dimensional to three-dimensional culture environments (37). Fibroblast-populated collagen lattices (FPLCs), which were initially developed to treat burns, have gained popularity as a model to study fibroblast integrin-mediated interactions with ECM substrate and associated paracrine biochemical signaling in three dimensions (5). These latices are seeded with fibroblasts and used to create either “free-floating” models, where the cells and their matrix float without adhesion to the surrounding experimental structures, or “rigid” models, where the matrix and fibroblasts are cast onto a fixed surface (38). Recent efforts to improve these models have focused on modulation of matrix porosity, stiffness, and adhesion domains to accurately represent the native fibroblast environment (37, 39, 40).

Computer-automated servohydraulic or vacuum-type stretch apparatuses allow for uniform frequency and amplitude of tension application to culture materials (38, 41–43). These apparatuses allow for specific modulation of parameters such as strain or compression magnitude, orientation, and kinetics (38). Traction is transduced by these apparatuses onto rings or two opposing bars which surround the matrix to which the fibroblasts are adhered (44–46). To apply mechanical force to individual cells, microneedles have been mounted on these apparatuses and inserted into the culture substrate in close proximity to fibroblast cells (47, 48). Use of “optical tweezers,” which consist of coherent light beams, have been used to trap, manipulate, and apply force to cells in a non-contact manner. These protocols have allowed for study of application of force to individual cells and study of mechanotransduction on a uni-cellular level (49–51). The development and implementation of atomic force microscopy has allowed for high-resolution and fluorescent live visualization of cells as they are stretched and undergo mechanical force (52–54).

### In vivo model systems

While *in vitro* systems have been the basis for a substantial portion of scientific literature to date examining the role of mechanical force on fibroblast behavior, they are limited in ability to replicate three-dimensional tension environments and biochemical crosstalk found in living tissue. *In vivo* models allow for a more holistic view of fibroblast activity within a dynamic tissue environment and represent a critical step in the scientific process of translating basic science findings into clinically applicable treatments.

Small animal models are a common first step for *in vivo* studies related to skin disease and offer the entry-level opportunity to study fibroblast activity in mammalian skin. While mouse skin lies more loosely than human skin, allowing for less scarring, it is genetically dissectible. To create a better analogue to human scarring in a mouse model, Arabi et al. developed a murine model of hypertrophic scarring that utilizes biomechanical loading devices that can be placed on opposite sides of a wound and distracted incrementally to apply continuous tension across the wound throughout the healing process. The scars resulting from this protocol were found to be histologically comparable to human hypertrophic scars (55). Similarly, Chin et al. developed a model using a computer-controlled device that distracts skin in a murine model cyclically to produce the effect of repetitive mechanical force on an area (56).

Porcine models provide several advantages over small animal models, such as higher anatomical fidelity to human skin compared to murine models. Additionally, porcine models provide scale and mechanical forces across the skin more similar to those found in humans. A hypertrophic scar model in pigs has been developed in which full-thickness, elliptical, and hexagonal excisions are created and put under tension by a load-bearing polymer device. This model produces hypertrophic cutaneous scars with histological morphology comparable to those found in human skin (57).

Advances in these model systems have allowed for great progress to be made in our understanding of the role mechanical forces play in fibroblast behavior and the wound healing process.

## Fibroblasts and myofibroblasts respond to and transduce tension

Dermal fibroblasts have long been implicated as the cells responding to mechanical force and affecting the clinical changes observed in scars under tension (5). These highly mechanosensitive cells have been found to respond to mechanical strain by increasing expression of proinflammatory and profibrotic genes, proliferating, migrating, and differentiating into myofibroblasts (5, 41, 58–60). While many pathways that contribute to fibroblast mechanotransduction in the skin are still in the process of being discovered and described, the past two decades have yielded increased depth of understanding into the cellular structures, genes, and signaling pathways by which fibroblasts respond to mechanical force and alter the wound environment (28).

In addition to responding to external mechanical force, fibroblasts have been shown to create mechanical forces within the wound to contract and assist with closure during the healing process. These mechanical forces are termed cell traction forces (CTFs) and are generated by the cytoskeleton of the cell (61, 62). This process is thought to underly the so-called “purse-string” healing model, mediated in adults by myofibroblasts formation of late-stage granulation tissue, which imposes contractile forces to pull the wound closed. Fibroblasts and myofibroblasts impose this intracellular tension through the sliding of ATP-powered actin-myosin filaments which is then transmitted to the ECM through focal adhesions at either end stress fibers. Actin polymerization at the leading edge of a moving cell serves as another means of CTF generation (63). The cellular motion associated with the creation of CTFs are relatively slow, sustained, and irreversible when compared to the calcium-regulated contraction of myocytes (64). A number of associated molecules regulate this process, including myosin light chain kinase (MLCK), MLC phosphatase, Rho, Rac, and α-SMA. It is through the generation of CTFs that fibroblasts and myofibroblasts are able to migrate within the ECM, generate stress and strain within tissue, maintain cellular tensional homeostasis, and ultimately draw wound edges together (64).

In normal wound healing, myofibroblasts facilitate contraction of the granulation tissue and then undergo normal apoptotic cell death. Normal wound healing concludes when the wound bed is re-epithelialized by keratinocytes which migrate to the wound edges *via* lamellipodia (65, 66). However, in cases of pathologic scarring, prolonged myofibroblast survival contributes to the aberrant fibrotic pathology (67–69). In fact, mechanical load has been shown to result in a four-fold decrease in myofibroblast apoptosis, which in turn results in a hypertrophic scar phenotype. This mechanism, in part, is thought to explain the cellular changes that underlie the pathogenesis of pathologic scarring (55).

## Mechanotransduction and biotensegrity

Although the concept of mechanical tension on wounds exacerbating scar formation has long been acknowledged, only recently have scientific studies allowed the cellular and physiological causes underlying this phenomenon to be uncovered. As this field of inquiry has emerged, the term “mechanotransduction” has been coined, referring to the methods by which mechanical forces are translated into biochemical signals (5).

In the framework of mechanotransduction, “mechanosensing cells” or “sensor cells” have the ability to recognize mechanical cues from the environment such as force, stress, strain, rigidity, and adhesiveness. Conversely, “mechanotransducing cells” or “effector cells” contain proteins or protein complexes that can produce or potentiate a chemical signal in response to mechanical stimulation (70).

**Figure 2:**
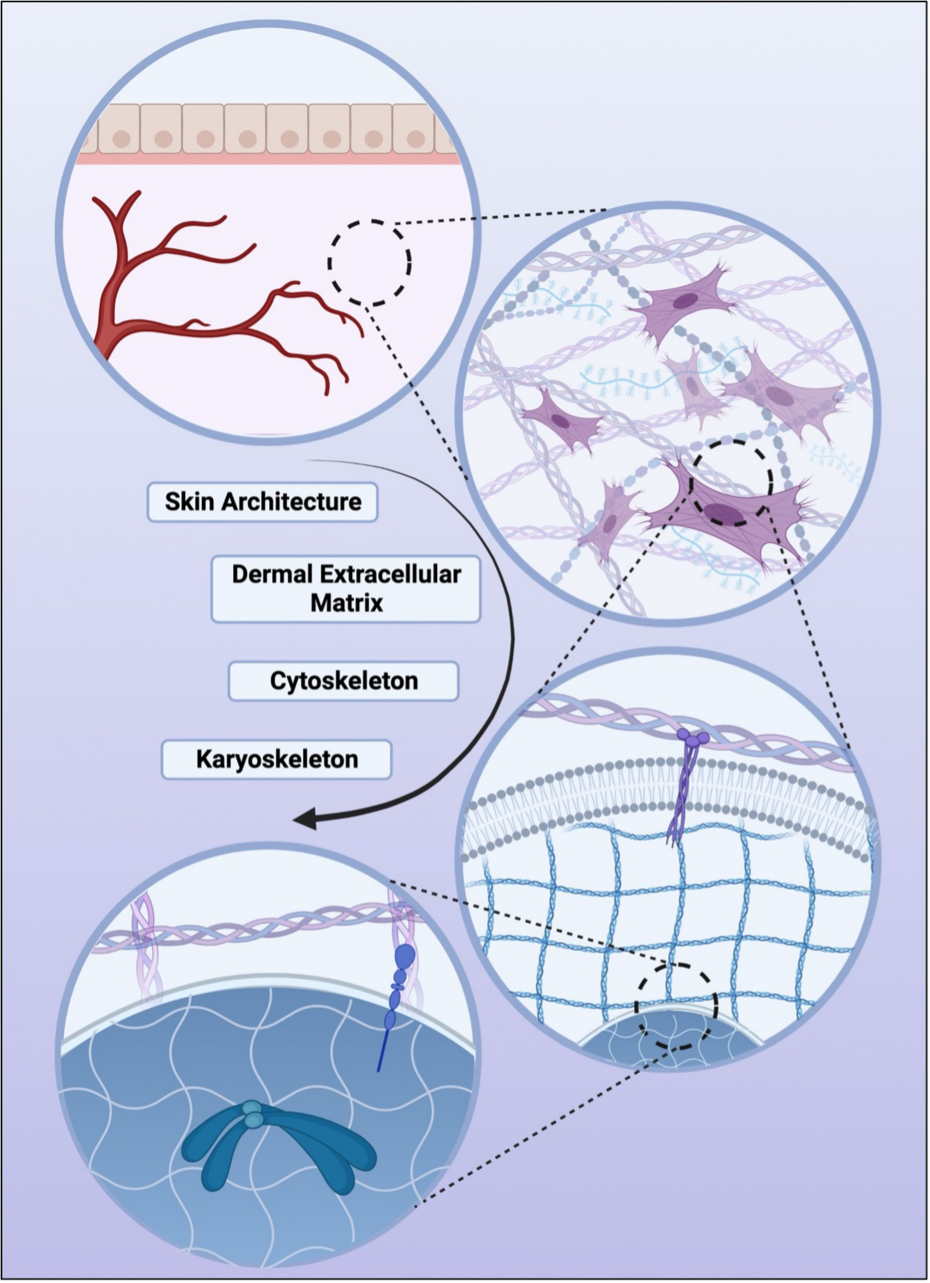
Various levels of biotensegrity, the structural principle which demonstrates how tissue, extracellular space, cell cytoplasm, and the nucleus are stabilized by continuous tension with discontinuous compression. In descending order, skin organ-level architecture, followed by the tissue-level dermal matrix, the cellular-level cytoskeleton, and finally the nucleus-level karyoskeleton. It is through these structural systems that mechanical forces are relayed to the cellular and nuclear level and one of the pathways through which mechanotransduction is thought to be facilitated.

Organs, tissues, cells, and sub-cellular components are understood to exist within a stabilizing and compressing framework which have broadly become known as the biotensegrity system. Coming from the architechtural term,“tensegrity,” biotensegrity refers to biological systems that are both stabilized by continuous tension and discontinuous compression. From a tissue level to a sub-cellular level, this framework allows mechanical forces to be relayed and therefore acts as an important mechanism in mechanotransduction (71). This framework is embodied by the ECM at a tissue level, the cytoplasm on a cellular level, and the karyoskeleton within the nucleus (Figure 2) (71, 72).

These concepts of mechanotransduction and biotensegrity work in tandem to allow for mechanical action to propagate to the cellular and subcellular level. While mechanical forces on the skin can originate at the tissue level, such as stretch initiated by muscles, these forces can also be mediated at the cellular level through cellular adhesions and cytoskeletal connections. Regardless of origin, external mechanical signals can be sensed by transmembrane cell surface adhesion receptors such as integrins, cadherins, or mechanosensitive ion channels which allow for transfer of mechanical stimulation across the cell plasma membrane. These mechanical forces are then able to be translated to intra-cytoplasmic biochemical signals by various means including cell focal adhesion complexes and intercellular adhesion complexes. Biochemical signals mediate downstream signaling cascades, and transcriptional effects in the nucleus. Therefore, the process of mechanotransduction can cause up- or down-regulation of second messengers and subsequently affect cell processes such as migration, growth, proliferation, and matrix remodeling (73). Signaling from mechanosensitive cells to mechanoresponsive cells results in the activation, suppression, and modulation of pathways key to tissue-level processes such as wound healing (74).

Mechanotransduction is understood to occur at a spatial distance when initial force acts across specific cytoskeletal filaments and the communication of this stimulation is known to depend on the stiffness differential between cellular structural components. Therefore, cytoskeletal prestress may in part determine the pace and fidelity of intracellular response to external mechanical signals. As these forces are transduced to the nuclear level, changes in the shape and kinetics of load-bearing molecules can result in epigenetic, transcriptional, and protein processing changes (75). These downstream effects of mechanical stimulation are highly relevant to cellular behavior, allowing for mechanotransduction to control cellular physiology and coordinate tissue-level impact (74, 76).

Modern developments in basic science techniques and computational ability have allowed for further characterization of fibroblast subpopulations which has yielded interesting and important insights into the behavior of fibroblasts in a cutaneous wound environment. For the past several years, fibroblast heterogeneity and interest in characterization of fibroblast subpopulations has grown. Foster et al. published a 2021 article detailing a multimodal-omics approach to study fibroblasts response to wound healing, using methods such as transgenic rainbow mouse lineage tracing, bulk transcriptomic analysis, and single cell RNA transcriptomic analysis. This study found that local fibroblasts proliferate in a linear, polyclonal manner along the cross-sectional wound interface (77). Foster et al. demonstrated that local fibroblasts migrate to the wound site following injury to the region with specific functional subpolulations involved in different processes. In particular, a mechanofibrotic subpopulation of fibroblasts exists on the outskirts of the wound, characterized by markers including Engrailed-1, COL1A1, TGFβ-2, and JUN. One week after the initial wound, these mechanofibrotic fibroblasts begin to proliferate in response to mechanical force and migrate to the center of the wound. By two weeks, fibroblasts maintained a fibrotic state in the scar microenvironment which was sustained by inflammatory signaling despite closure and epithelialization of the wound.

## Cellular mechanisms of mechanotransduction

### Extracellular mechanisms and integrins

Cells exist within a protein-rich scaffold known as the ECM, which provides structural support and mediates connections between cells. The ECM plays important roles in the wound healing process, including modulation of biochemical signaling pathways and regulation of the proliferation, migration, and survival of cells existing within it (78). While the ECM is made up of over 300 protein and polysaccharide types, the most frequently found are collagen and elastin, which are morphologically string-like and attach to cells *via* transmembrane heterodimeric receptors known as integrins (79, 80). These proteins are fundamental to transmission of signals from outside of the cell inwards and vice versa.

Integrins connect the ECM to the intracellular actin cytoskeleton and are important components of focal adhesions, which unsurprisingly have long been an area of interest for researchers interested in mechanotransduction (5). Focal adhesions are composite structures of several proteins which attach the intracellular cytoskeleton to the extracellular matrix. When a mechanical force acts upon the extracellular matrix, these complexes mediate the propagation of the mechanical signal into downstream biochemical pathways. These biochemical pathways ultimately result in the migration of inflammatory cells and keratinocytes, initiation of angiogenesis, and increase in collagen synthesis that causes scarring (81).

### Transforming growth factor Beta (TGF-β)

Transforming growth factor β (TGF-β), a multifunctional growth factor and one of the most well-described pro-fibrotic cytokines, works in multiple pathways to translate mechanical stimulation to fibrotic pathology. There are three primary isoforms of TGF-β: TGF-β_1_, TGF-β_2_, and TGF-β_3_, all of which contain a heteromeric receptor complex of type I and II receptor serine/threonine kinases (82). TGF-β first binds with the TGF-β receptor 2 (TGF-βR2) before the signal is propagated downstream through Smad family proteins, which can be categorized into receptor-regulated Smads or R-Smads (Smads 1, 2, 3, 5, and 8), common-partner Smads or Co-Smads (Smad 4), and inhibitory Smads or I-Smads (Smads 6 and 7). R-Smads for a hetrodimeric complex with Co-Smads before translocating to the nucleus to act as a transcription factor. Classic transcriptional targets of the TGF-β pathway have pro-fibrotic effects such as the induction of excessive collagen production and the initiation of fibroblast transition to myofibroblasts (83–85).

TGF-β plays a central role in fibroblast mechanotransduction pathways. In the fibroblasts of hypertrophic scars, the autocrine production and activation of TGF-β results in the development and stabilization of large focal adhesions and upregulates myofibroblast contractility which are thought to contribute to the excessive contraction of wounds seen in this pathology (86, 87). TGF-β has also been shown to upregulate the fibroblast contractile markers *α*-smooth muscle actin (*α*-SMA), cofilin, and profilin in myofibroblasts in a dose-dependent manner (88, 89).

TGF-β has been shown to be released and activated from its reservoir in the ECM due to integrin stimulation by mechanical force. However, Wipff et al. demonstrated an interesting effect of myofibroblast contraction to activate TGF-β from the ECM reservoir. Therefore, both external mechanical force and intrinsic contraction of myofibroblasts (which is itself stimulated by TGF-β) can result in the release and activation of TGF-β (90).

### Focal adhesion kinase (FAK)

Focal adhesion kinase (FAK) was first recognized in 1992 as a non-receptor tyrosine-phosphorylated protein which localizes to focal adhesions and has quickly risen in interest as a component of mechanotransduction and a possible target in its inhibition. Several binding sites specific to focal adhesion proteins are present in the c-terminal domain of FAK that associates with integrin clusters. Integrin-dependent autophosphorylation of FAK at the Tyr-397 site and others is thought to activate kinases in the Src family which in turn initiate downstream signaling (47).

FAK's role was first characterized in relationship to cell motility, and in the early 2000s, *in vitro* studies began to explore the potential role of FAK in mechanotransduction. Wang et al. demonstrated that FAK-null fibroblasts showed impaired response to mechanical input during migration (47). Other studies have reported FAK phosphorylation followed by mitogen-activated protein kinase (MAPK) activation can be induced by uniaxial cyclic stretching of fibroblasts and resulted in fibroblast proliferation (41, 91). FAK has also been implicated in mechanotransduction pathways sensing shear stress in the endothelial cells of vasculature (92, 93).

In 2011, Wong et al. reported that following cutaneous injury, FAK is activated in a pathway potentiated by mechanical stimulation. In a murine model of hypertrophic scar formation, this study showed that FAK-knockout mice formed scars with less inflammation and fibrosis than control mice (38). Additionally, this research established extracellular-related kinase (ERK) as an important mediator of FAK. When wounds are under tension, ERK mediates the excessive production of collagen and triggers the release of the chemokine monocyte chemoattractant protein-1 (MCP-1). MCP-1 knockout mice formed minimal scars, and small molecule inhibition of FAK in human cells also reduced scar formation and attenuated MCP-1 chemokine signaling *in vivo* (94). This research established the role of the FAK–ERP–MCP-1 pathway as a key player in mechanotransduction and represented the beginning of identification of specific biochemical targets for uncoupling mechanical force from biochemical stimulation of pathologic scarring.

In 2022, Chen et al. published research utilizing these findings in a porcine model of split-thickness skin-grafting (STSG), a common intervention for deep tissue injuries that is also associated with causing contractures and scarring (95). Using single cell RNA analysis, the group found that STSGs indeed cause upregulation of proinflammatory and mechanotransducive pathways as would be expected in scar formation. A FAK inhibitor was applied to this model, and found to promote healing, reduce contracture, mitigate scar formation, restore collagen architecture, and improve graft biomechanical properties. Single cell RNA analysis indicated that application of a FAK inhibitor up-regulated myeloid CXCL10-mediated anti-inflammatory effects and decreased CXCL14-mediated chemokine action and fibroblast migration. Mechanical force was found to increase fibroblast transcription of pro-fibrotic genes at later timepoints and interruption of mechanical stimulation by FAK inhibition resulted in a shift toward pro-regenerative fibroblast states that typically characterize unwounded skin (95).

### Rhoa and rho-associated kinase (ROCK)

Perhaps the best described target of FAK signaling, the Rho family of GTPases have been demonstrated to influence fibroblast behavior such as tension, motility, intercellular-adherence, cytoskeletal dynamics, and differentiation into myofibroblasts. In a murine model of cardiac injury, a knockout of Rho-associated kinase-1 (ROCK-1) resulted in lower levels of myofibroblast transition in response to ischemia (96). Cyclic mechanical tension has been shown to activate RhoA and cause induction of ROCK-dependent actin assembly, while the cytoskeleton was relaxed with use of a ROCK inhibitor (59). RhoGTPases also appear to have a connection to effectors of the Hippo pathway, which has been connected strongly to cell proliferation, apoptosis, differentiation, and malignant transformation (97, 98).

### Hippo pathway: yes-associated protein (YAP) and transcriptional coactivator with PDZ-binding motif (TAZ)

In 2011, Dupont et al. identified that the two main downstream effectors of the Hippo pathway, Yes-associated protein (YAP) and transcriptional coactivator with PDZ-binding motif (TAZ), are involved in nuclear transduction of mechanical signals occurring in response to changes in ECM rigidity and shape (99). This pathway was found to be Rho GTPase-dependent and required tension on the actomyosin cytoskeleton but was found to occur regardless of Hippo signaling. Additionally, this study implicated YAP and TAZ as important regulators of cellular differentiation, survival, and regeneration based on ECM stiffness (99, 100).

Lee et al. expanded on this finding withing wound healing in a 2014 murine model of cutaneous scarring. YAP was found to localize to the nucleus of dermal cells at 2- and 7-days following wounding. TAZ, which normally localizes to the cytoplasm of dermal cells, localized to the nucleus as well one day following wounding. In YAP/TAZ knockdowns, the rate of wound closure was markedly slowed and TGF-β expression was reduced. Additionally, Mascharak et al. demonstrated YAP inhibition to alter fibroblast behavior preventing adaption of a more profibrotic phenotype and resulting in regeneration of skin following wounding as opposed to scar formation (101). The mechanosensitive proteins YAP and TAZ have been found to modulate many molecules important for the development of fibroproliferative diseases such as scarring, including proteins in the TGF-*β* signaling pathway, such as Smad-2, Smad-7, and p21, as well as connective tissue growth factor (CTGF) and transglutaminase-2 (99, 102). The results of this study support that the Hippo pathway, and particularly YAP and TAZ, is essential for dermal wound healing (103).

YAP inhibition has been shown to block activation of engrailed-1 expression, leaving fibroblasts in a regenerative state to promote wound regeneration (104). Multiomic analyses have further elucidated these divergent molecular trajectories, with inhibition of YAP mechanotransduction to drive fibroblast mediated regenerative repair through TRPS1 and Wnt activation (101).

### Wnt-**β**-Catenin

Notably, connections have been drawn between the Hippo pathway and components of the canonical Wnt-β-Catenin pathway (105). A 2002 study initially linked β-catenin to fibroblast activity in cutaneous wounds, when Cheon et al. showed that the core component of the cadherin protein complex stabilized fibroblast proliferation, motility, and invasiveness in cutaneous wounds. Transgenic mice with elevated β-catenin activity developed hyperplastic scars following cutaneous wounding (106). Since then, several studies have shown that β-catenin is mechanoresponsive and mediates myofibroblast differentiation, though the significance of this pathway to cutaneous wound healing has yet to be elucidated fully (107, 108).

### Calcium channel Ion pathways

Calcium channels are one of several ion channel types that allow for maintenance of an electrical and chemical gradient across the plasma membrane of cells. Creation of these gradients and subsequent depolarization of ions across them can result in second messenger activation and a variety of downstream effects. Because certain types of calcium ion channels can be activated by mechanical force, they provide a means of mechanotransduction of signals from the outside of the environment to the intracellular space (109). This has been shown experimentally to occur in fibroblasts, with intracellular levels of calcium elevating in response to application of hydraulic pressure or stretch (48, 110). The action of these channels have been shown to be intimately related to the physical connections between integrin and the intercellular actin cytoskeleton, indicating important relationships between calcium homeostasis and cellular mechanical sensing (74).

One theory of the mechanism behind coordinated myofibroblast contraction facilitating wound closure postulates that signaling through adherens junctions to nearby cells may result in the opening of mechanosensitive ion channels and a Ca^2+^ influx (74, 111).

### Piezo1

**Figure 3:**
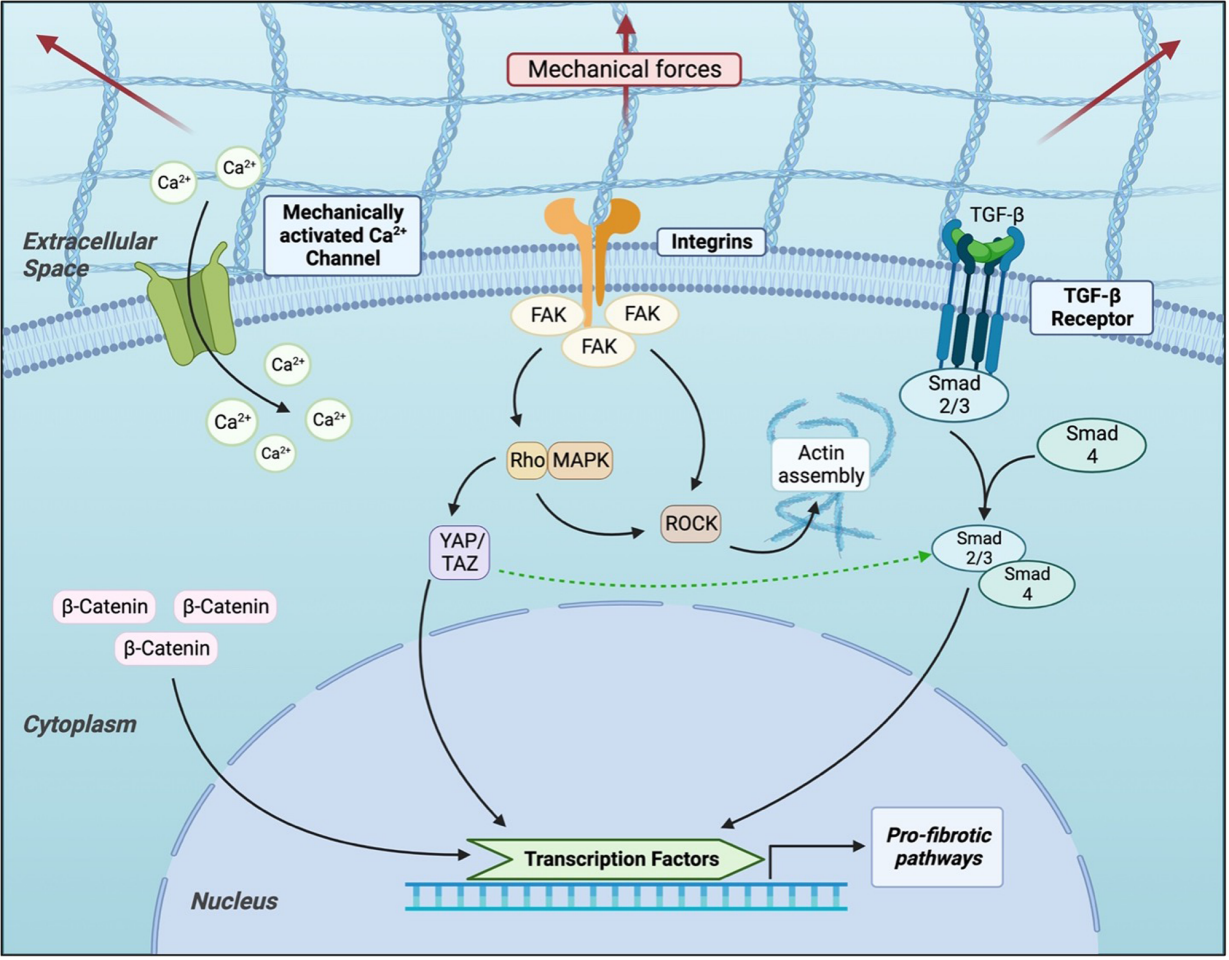
Current understanding of cellular signaling pathways related to mechanotransduction in fibroblasts.

In 2010, Coste et al. identified the two proteins Piezo1 and Piezo2 as significant components of mechanically activated cation channels (112). Following this discovery, a group of subsequent studies sought to characterize the role of these proteins. Nourse and Pathak demonstrated in 2017 that Piezo1interacts with the cell cytoskeleton, and in 2021 He et al. found that myofibroblasts in hypertrophic scar tissue overexpress Piezo1 (109). An *in vitro* model of mechanical stretch was shown to increase Piezo1 expression and calcium influx to human dermal fibroblasts, mediated by Piezo1. This model also demonstrated that Piezo1 activation promoted human dermal fibroblast proliferation, migration, and altered response to mechanical force, and Piezo1 inhibitor GsMTx4 was injected intradermally into rats, they were protected from mechanical force-induced hypertrophic scar formation (Figure 3) (113).

## Pathogenesis of keloids

As basic science techniques have continued to characterize the action of fibroblasts in the wound microenvironment, researchers have asked how these findings might apply to the case of keloids. Keloids are, as has been described above, one of the clearest clinical examples of tension affecting scar formation, and many have hypothesized that fibroblast mechanics might play a critical role in this process. Keloids have been characterized as a uniquely human example of pathologic scarring with various causal factors, though genetic factors appear to be the most influential. Several studies have examined the roles of apoptosis inhibition, nutritional factors, sebum secretion, chronic inflammation, and neurogenic inflammation as factors contributing to keloid pathogenesis (28).

Unsurprisingly, mechanical forces on fibroblasts have indeed been found to act significantly in keloid pathogenesis. When fibroblasts present in unwounded skin are compared to those in keloid scars, stiffness and force generated by the cell's actin filaments increased (114). These findings are hypothesized to contribute to the ability of keloid fibroblasts to migrate outside of the original wound limits. A 2018 study by Hsu et al. identified that decreased expression of caveolin-1, a cellular membrane protein, could contribute to increased flexibility of the membrane and resulting aberrant responsiveness of keloid fibroblasts to mechanical stimulation. Perhaps for this reason, keloid fibroblasts were found to produce excessive levels of pro-fibrotic cytokines and ECM when cultured on a mechanically stiff substrate. This action was mediated by nuclear translocation of Runx2, which is a transcription factor related to osteogenesis (115). Keloid fibroblasts have also been shown by Wang et al. to produce higher levels of TGF-β1, TGF-β2, and collagen 1a at both the transcriptional and translational levels when exposed to equiaxial strain. Additionally, keloid fibroblasts generate more focal adhesion complexes and demonstrate increased activation of FAK when exposed to mechanical stimulation (116).

The past two decades of research have expanded the body of knowledge related to keloid pathogenesis and the causes of pathologic scarring generally. As these studies have continued to implicate mechanical forces as an important mediator of these pathologies, translational clinical treatments have begun to emerge with this target in mind.

## Current clinical options for treatment and prevention of pathologic scarring

The body of research detailed above describes how mechanical force on the wound contributes to formation of scar tissue as wounds heal. With the goal of translating this important scientific finding to clinical medicine, several clinical interventions have emerged to prevent and treat scarring through attenuation of mechanical force on healing wounds.

### Botulinum toxin A

Derived from the bacteria Clostridium botulinum, botulinum toxin A provides neurotoxic effects which can halt neuromuscular transmission and therefore decrease tension applied to skin by underlying muscular movement. Due to the ability of botulinum toxin A to paralyze the muscle and therefore decrease tension on the edges of wounds, interest has arisen in the ability of this agent to treat pathologic scars that are known to be exacerbated by mechanical forces, such as keloids and hypertrophic scars (57). Indeed, studies have demonstrated that treatment of muscular structures surrounding a wound with botulinum toxin A decreases fibroblast proliferation and ultimately leads to decreased expression of TGF-β1 (117). Clinically, the application of botulinum toxin A to wounds and the surrounding area, typically *via* intralesional injection, has been reported to reduce scar formation. A recent meta-analysis demonstrated that keloids and hypertrophic scars treated with botulinum toxin A appeared visually less noticeable than those treated with corticosteroid or placebo (118). Prospective clinical studies have reported elevated levels of patient satisfaction with botulinum toxin A treatment, as well as decreases in pain, pruritis, tenderness, and scar volume (7, 118–120).

### Silicone gel sheeting

External dressings placed on wounds during healing can help limit mechanical forces like stretching. Silicone gel sheeting, in particular, has been used since the 1980s in the clinical treatment of hypertrophic scars and keloids. Clinical studies have shown visible improvement in scars with silicone gel sheet application and analytical software has demonstrated that this material facilitates the transfer of tension from the wound to surrounding normal skin (15, 121, 122). However, some attribute these positive clinical outcomes to the silicon gel's ability to hydrate the stratum corneum and subsequently prevent fibroblast proliferation and collagen deposition (122, 123). Regardless of mechanism, silicone gel and other stabilization dressings remain a popular clinical prophylactic for scar formation, due to consistently favorable performance in randomized control clinical trials and exhibition of only mild side effects such as skin irritation (15, 124).

### Tapes

In the context of hypertrophic scarring, taping has been used clinically to aid in opposing the edges of a fresh wound during closure, but recent interest in the usage of tape to reduce tension across the incision during healing has risen. Types of tapes used clinically range in attributes, including non-stretch, paper, and elastic (high-stretch) varieties.

#### Non-stretch tape

Current literature has demonstrated the most support for non-stretch tapes, such as Blenderm™ in the prevention of scarring following wounds. 2021 systematic review found that non-stretch tapes reduced the height, width, color, associated itching, and gross visual score associated with scars when implemented early in the treatment course (125). When implemented at later time-points, non-stretch tapes were found to have a high level of evidence for improving thickness, pliability, softness, and color of scars (125, 126). Non-stretch tapes have also been implemented, with mixed results, in the treatment of burns and for use in scar hydration (125, 127).

#### Paper tape

Paper tapes, such as Steri-Strips™ and Micropore™, have long been used clinically, particularly in the early stages of wound management. Indeed, studies suggest that these materials prevent hypertrophic scarring when implemented in early treatment, though some studies suggest that hypertrophic scarring or scar stretching may occur after the removal of paper tape 12 weeks following injury (128, 129). One paper found that use of paper tape reduced pain, itch, thickness, and elevation of the scar 12 months following initial wound. Generally, paper tapes have been found to have less efficacy when applied during the remodeling phase of wound healing or on mature scars, though have been reported to demonstrate subjective improvements in scar coloration, thickness, and elasticity (125, 126).

#### Elastic tape

Elastic-type tapes such as Kinesio tape are infrequently used in the acute treatment setting, but have been reported in a single case study to produce an improvement in color, pliability, and elasticity in the late treatment of hypertrophic scarring (125). Additionally, 70% of patients reported improved satisfaction with the appearance of their previously untreated mature scars when elastic tape and zero stretch was applied (130, 131).

### Oyster splints

Keloids classically appear often on the lobes of the ears following trauma to the area, creating a functionally and aesthetically undesirable result. The oyster splint was developed to treat keloids of the ear in 1983 by applying compression to the area subsequent to surgical correction of the keloid (132). As the ear presents a challenge to apply compression to due to topographical irregularities and anatomical heterogeneity, the oyster splint requires a mold of the area to be made and compressive pressure is then applied to a splint in the unique shape of the patient's wound and ear. The oyster splint reduces tissue metabolism and fibroblast proliferation, likely related to pressure-offloading from the wound margins (133). Case reports have suggested that this technique results in favorable functional and aesthetic clinical outcomes compared to scar revision surgery alone (132, 133).

### Embrace device

Designed with the specific goal of offloading tension from a wound and subsequently improving aesthetic and functional outcomes following scar revision surgery, the Embrace device is a pre-strained silicone elastomeric dressing which is adhered to the skin with pressure-sensitive silicone adhesive. A clinical study where this device was applied to one half of a scar revision incision in 10 patients found a highly significant improvement in scar appearance with treatment, suggesting promise for such technologies in treating pathologic scarring (134). A second randomized control trial treating one half of an abdominoplasty closure using the embrace device and the other half with the surgeon's standard of care also found significantly reduced scarring (135). Future opportunities involving this device may include customizing usage and design of the device for specific anatomical areas and incisions.

### Incisional negative pressure wound therapy (iNPWT)

Use of iNPWT has been implemented previously to prevent infection and dehiscence, a 2022 study investigated the use of this technology to study the effect on scarring. The hypothesis behind this work considered that iNPWT may increase lateral tension across the incision, ultimately decreasing mechanical pulling forces that contribute to hypertrophic scar formation. In incisions following gender-affirming mastectomies, one randomized side was treated with iNPWT while the other was treated with Steri-Strips^TM^. While objective quantitative results were not remarkably different between the two groups, iNPWT resulted in improved patient satisfaction results on SCAR-Q and the PSOAS Observer scale (136).

### Sutures

To reduce the risk of pathological scar formation, intentional use of suture types and placement which reduce tension across a surgical wound are preferrable. Specifically, attention is often paid to tension across the wound dermis, given evidence of the importance of this layer in the formation of keloids and hypertrophic scars (6). This logic, in part, has helped popularize the technique of using subcutaneous tensile reduction sutures which intentionally displace tension from the dermis to the deep fascia and superficial fascia layers (5).

### Scar revision techniques to reduce tension

When mature scars occur that result in functional or aesthetic concerns for a patient, the traditional treatment is surgical scar revision. Importantly, the remodeling phase of wound healing can continue for one or more years after initial injury and therefore surgical intervention should be considered after this timepoint, as scars may regress naturally. The goal of these procedures is to alleviate functional limitations caused by the scar tissue or improve the visual appearance by removal of scar tissue or utilizing anatomical geometries to reframe the scar and decrease noticeability. Surgical intervention is effective for treating hypertrophic scars, which recur at low rates, but should be used conservatively in the treatment of keloids, which recur at rates estimated at 45%–100% (137–139). A critical pillar of scar revision is tension-free closure, which can be facilitated by surgical technique (6, 140). The z-plasty technique, heralded for its ability to lengthen contracted scars and align scars more smoothly with skin tension lines, has been popularized in the setting of scar revision (5, 141, 142). In some cases, local flaps can be utilized to decrease tension on the wound and ultimately assist in resolving pathologic scarring (143).

## Conclusion

Scarring of the skin poses a complicated clinical problem with significant implications for patient functional and psychological outcomes and with large socioeconomic impact. It has long been recognized that tension and force across a wound affects scar formation, particularly in the context of pathologic scarring. Therefore, scientific endeavors have long attempted to better characterize the process of mechanotransduction and how mechanical force is translated to changes in cellular biochemical signaling, in cutaneous wounding. Using a variety of inventive *in vitro* and *in vivo* models, scientists have demonstrated the importance of the fibroblast as a critical cell in mechanosensitivity and mechanoresponsiveness. Several proteins, transcriptional cofactors, and signaling pathways, have been characterized in connection to mechanotransduction in cutaneous injury, and novel technologies in cellular biology have allowed for more granular understanding of these interactions and fibroblast behavior in response to mechanical stress than ever before.

When a wounded area is under tension, a larger scar is known to form. The larger the fibrotic area caused by the healing scar, the stiffer the environment becomes. In this way, a “vicious feedback loop” is created wherein mechanical tension results in continuously stimulated fibroblast overactivation and excessive production of ECM which can subsequently progress to pathologic scarring and contraction (113). As we continue to understand fibroblast behavior in the wound microenvironment, we grow closer to identifying targets for translational therapies for pathologic scarring. Mechanotransduction continues to be identified as a critical mediator of scar formation and may well hold the key to scarless healing.
